# Fighting for Chemicals: Pharmacophagy‐Induced Contest Behaviour and Its Molecular Signatures in a Sawfly

**DOI:** 10.1111/mec.70345

**Published:** 2026-04-15

**Authors:** Sarah C. Paul, Alice B. Dennis, Lisa Johanna Tewes, Jeanne Friedrichs, Pragya Singh, Caroline Müller

**Affiliations:** ^1^ Chemical Ecology Bielefeld University Bielefeld Germany; ^2^ Unit of Evolutionary Biology/Systematic Zoology Institute of Biochemistry and Biology, University of Potsdam Potsdam Germany; ^3^ Adaptive Evolution and Genomics Institute of Life, Earth, and Environment, University of Namur Namur Belgium; ^4^ Joint Institute for Individualisation in a Changing Environment (JICE), University of Münster and Bielefeld University Bielefeld Germany

**Keywords:** aggression, chemical defence, contest behaviour, gene expression, pharmacophagy

## Abstract

Elucidating the multifaceted effects of plant metabolites sequestered by animals from plants for non‐nutritional purposes, known as pharmacophagy, is fundamental to understanding the evolution of acquired chemical defences. Some herbivorous species do not only take up plant metabolites from plants but also engage in antagonistic interactions to acquire precious metabolites from conspecifics that had access to the plant chemicals. We investigated behavioural and molecular consequences of pharmacophagy using the turnip sawfly, *Athalia rosae*. Adults are known to be attracted to the plant *Ajuga reptans* . Using behavioural assays and chemical analysis, we showed that the sawflies acquire clerodanoids by licking the leaves. Moreover, aggressively licking conspecifics that had acquired plant clerodanoids resulted in the transfer of these metabolites between individuals. Transcriptomic analyses revealed that the acquisition of clerodanoids from leaves led to only minimal upregulation of known detoxification genes and pathways. In contrast, the aggressive licking of conspecifics resulted in the upregulation of metabolic pathways associated with elevated energy consumption or detoxification. Thus, individuals attack conspecifics to acquire clerodanoids despite the apparent metabolic costs. Changes in the metabolic phenotype of *A. rosae* individuals have profound consequences for the individuals' physiology and interactions with conspecifics, with possible impacts on the social niche.

## 1 Introduction

Herbivores not only take up plant metabolites for nutritive purposes, they can also acquire specialised metabolites from plants and store them for nonnutritive purposes, a phenomenon also known as pharmacophagy (Boppré 1984; Singh and Müller 2025). These metabolites can be used for defence against predators (Rojas et al. 2017), self‐medication against parasitoids or microbes (Abbott 2014; Tan et al. 2019), or for pheromone synthesis (Reddy and Guerrero 2004). By acquiring and occasionally modifying plant metabolites, individuals alter their chemical phenotype with potential ramifications for interactions with conspecifics and therefore their social niche (Kaiser et al. 2024; Müller et al. 2020). In some cases, these acquired plant metabolites are further distributed within conspecifics, often via nuptial gifts (Hashimoto and Hayashi 2014; Wink 2019). However, the metabolites may also be taken from a conspecific in agonistic ways, for example, if the plant itself is not available. In that way, pharmacophagy may have different ‘social dimensions’.

Competition between individuals over resources such as food (Jakob 1994) or mates (Takeuchi 2017) is well known, but competition between individuals over plant metabolites acquired by pharmacophagy is comparatively less explored. Because pharmacophagously acquired phytochemicals can confer significant fitness benefits (Reddy and Guerrero 2004; Rojas et al. 2017; Tan et al. 2019), their sources may become an arena for contest, especially when there is chemical inequality, with some individuals possessing and others lacking the chemicals, for example, if the sources are scarce or patchily distributed. Following predictions from game theory, the occurrence and outcome of contest interactions between two conspecifics depends on the balance between the costs and benefits of engaging in a fight for each individual (Arnott and Elwood 2009; Enquist and Leimar 1987). This balance is closely linked to an individual's likelihood of winning, or its resource holding potential (RHP), and the value of the contested resource to each competitor, referred to as resource value (RV) (Arnott and Elwood 2009). RHP is commonly determined by the size of an individual and its condition (Vieira and Peixoto 2013). For example, larger competitors are more likely to win a fight against smaller or less experienced individuals (Chamorro‐Florescano et al. 2011). In terms of RV, for example, females with offspring are more likely to win contests against males over burrows than females without offspring (Figler et al. 1995). When competing for phytochemical resources, an individual with no or low levels of the chemical may win the interaction if differences in RHP are minimal. The costs of fighting, such as increased energetic expenditure (Hack 1997) and predation risk (Jakobsson et al. 1995), will be outweighed by the fitness benefits of acquiring potent phytochemicals.

Pharmacophagy itself can carry costs, particularly when toxic plant metabolites are sequestered (Ruxton et al. 2018). The extent to which sequestration is costly varies considerably between species (Züst et al. 2018; Zvereva and Kozlov 2016), suggesting that these costs likely depend on the nature of the metabolite(s) being obtained and on the organism's physiological mechanisms for processing them (Heckel 2014). Key enzymes such as cytochrome P450 monooxygenases (P450s), glutathione S‐transferases (GSTs), UDP‐glucuronosyltransferases (UDPs), and ABC transporters are involved in detoxification and sequestration by herbivores, while heat shock proteins (HSPs) are typically expressed under a wide variety of stressful conditions, including those requiring detoxification (Xu et al. 2013). Understanding the mechanisms underlying sequestration costs in contest scenarios is crucial, as this may inform an individual's motivation to fight (RV) over pharmacophagously acquired resources. An individual that has already obtained some chemicals may still seek access to a resource if the effects of the chemical(s) are concentration‐dependent (Burdfield‐Steel et al. 2019). In contrast, an individual that has collected larger amounts of metabolites by pharmacophagy may have reached a threshold of sequestration costs or an adequate level of the metabolites.

Adults of *Athalia rosae* (Hymenoptera: Tenthredinidae) pharmacophagously acquire neoclerodane diterpenes (hereafter called ‘clerodanoids’) from non‐food plants such as *Ajuga reptans* (Lamiaceae) (Nishida et al. 2004), by licking the plant surface with their maxillary and labial palps, sometimes followed by a ‘body‐lotioning’ behaviour (Brueggemann et al. 2025). Uptake of these metabolites alters their metabolic phenotype, and clerodanoids are detectable on the insect surface (Brueggemann et al. 2023). This change has positive effects on both female mating success and predator defence (Amano et al. 1999; Müller 2009; Singh et al. 2022). Like many other pharmacophagous species, *A. rosae* adults also slightly modify the metabolites that they acquire (Brueggemann et al. 2023; Nishida et al. 2004). However, what makes them unique is that in addition to licking (previously referred to as ‘nibbling’) leaves, adults try to lick conspecifics in an aggressive manner to acquire these metabolites from the body of their opponent, impacting their social interactions (Singh et al. 2024).

Here we aimed to document this agonistic licking behaviour between males and to test whether (1) it is stimulated by a conspecific opponent's acquisition of plant clerodanoids and if it varies with an individual's own clerodanoid status, (2) it results in the transfer of metabolites between individuals, and (3) it leads to differential gene expression between individuals that collect clerodanoids from leaves or exclusively from conspecifics. Specifically we predicted that (1) the strongest determinant of successful licking would be the disparity between individuals in clerodanoid acquisition (‘chemical inequality’), that is, individuals lacking clerodanoids would lick more when exposed to individuals that had already acquired these compounds; (2) licking would result in the transfer of metabolites between individuals; and (3) pharmacophagy on leaves would result in the upregulation of canonical detoxification and sequestration pathways and genes, whereas licking exclusively conspecifics would induce the upregulation of metabolic pathways related to increased energy production and stress.

## 2 Materials and Methods

### 2.1 Insect Rearing

Adults of *A. rosae* were collected in a meadow in Verle, Germany (51°52′23.0″N 8°33′32.0″E), and kept in two cages. They were provided with *Sinapis alba* plants for oviposition and *Brassica rapa* var. *pekinensis* plants as food for larvae. Larvae of the F2 generation were transferred into individual pots with soil for pupation. After emergence, adults were placed individually in Petri dishes (60 × 15 mm) with honey‐water (1:50 v:v)‐soaked tissue paper as a food supply. Adults were kept at ~8°C until their use in contest bioassays. Rearing took place in a climate chamber with a 16:8 h light:dark cycle at 20°C (light) and 16°C (dark), and 70% r.h. Host plants were grown from seed in a greenhouse (no climate control, light:dark 16:8 h).

### 2.2 Contest Bioassays

**FIGURE 1:**
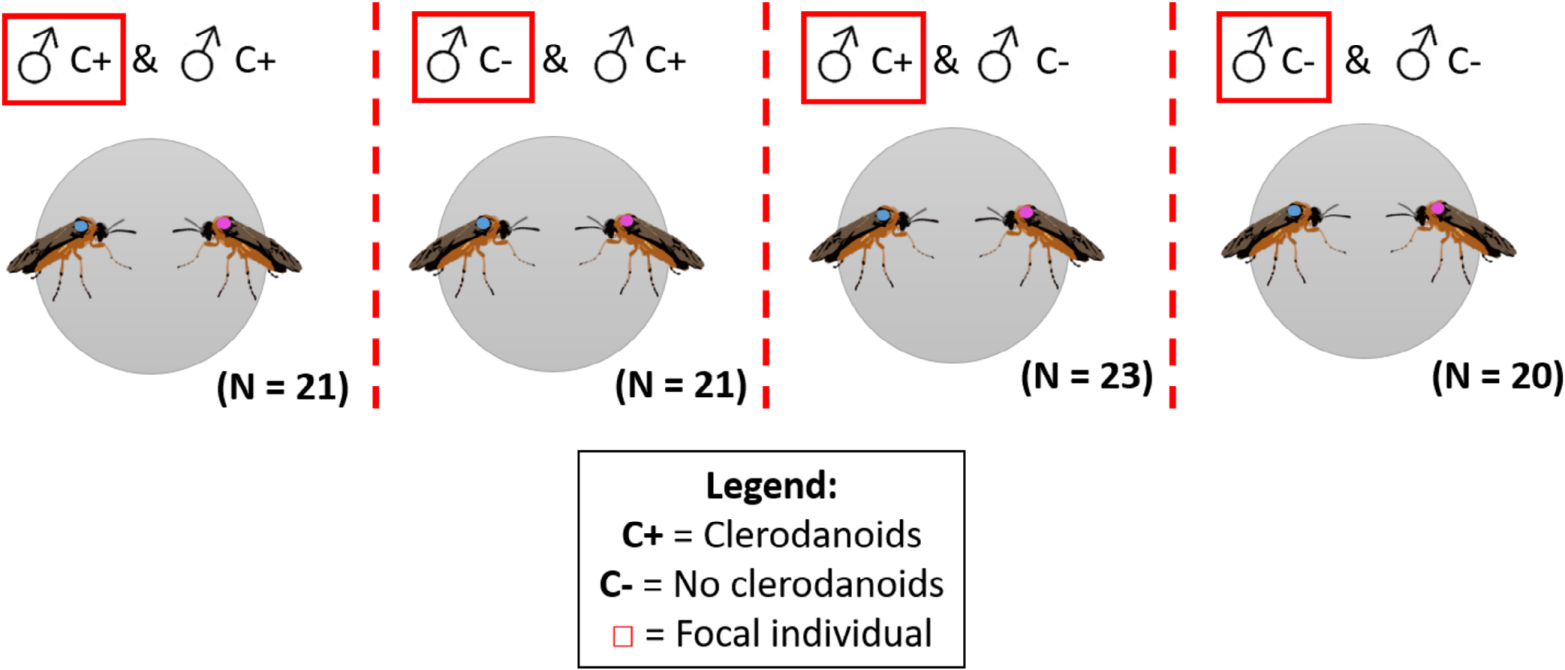
Overview of the treatments and number of pairs used (N) in the male–male contest assays of *Athalia rosae*. C+ individuals (with clerodanoids) were provided with a leaf of *Ajuga reptans* 48 h prior to the assays, whereas C– individuals (no clerodanoids) were not.

Contest bioassays were conducted on pairs of male *A. rosae* across four treatment levels defined by focal and opponent clerodanoid acquisition status [i.e., with clerodanoids taken up from plants (C+) or without clerodanoids (C–); Figure 1], avoiding the pairing of siblings. We focused on males to exclude impacts of different reproductive resources; for example, females may contain different numbers of eggs, which could influence behaviour and physiology and confound effects of clerodanoid acquisition. Two days prior to the bioassay trial, adults (3–14 days post‐eclosion) were removed from the refrigerator, weighed to the nearest 0.01 mg (Sartorius AZ64, M‐POWER Series Analytical Balance), provided with a honey–water mixture, and individuals in the C+ treatment were additionally provided with a small piece (1 cm^2^) of a leaf of *A. reptans* . Plants of this species had been collected close to a forest (52°01′58.2″N 8°29′04.5″ E) and were kept outdoors. Males were marked on the thorax with quickly drying lacquer (Maybelline Colour Show 60Seconds, Maybelline, New York) in either pink (16R 200) or blue (16R 400). The marking colour of the focal individual was alternated within each treatment level between trials, and individuals were matched for size (based on their mass).

Two individuals were added to opposite sides of a Petri dish and filmed for 25 min (Sony HDR‐CX410VE Camcorder, AVCHD—1920 × 1080–30 fps). At the end of each trial individuals were isolated, provided with fresh honey–water mixture and kept for another 48 h to allow for potential metabolism of any clerodanoids gained through licking. Subsequently, they were frozen at −80°C until chemical analysis (see below).

The software BORIS v.7.9.7 (Friard and Gamba 2016) was used to analyse the video data, with the analysis conducted by a single observer blind to the treatment. Successful licking behaviour was classified as the mouthparts of one individual making contact with the body of another individual and its occurrence and duration were recorded at 0.3× the original speed and at 2× display magnification.

### 2.3 Analysis of Pharmacophagy and Conspecific Transfer of Clerodanoids

To test whether licking *A. reptans* leaves resulted in the acquisition of clerodanoids by *A. rosae* , we chemically analysed C+ (*n* = 8) and C– (*n* = 7) individuals from the contest trials (C+C– and C–C– treatments) that had not engaged in any conspecific licking. To test whether conspecific licking resulted in the acquisition of clerodanoids, C– individuals that had licked C+ individuals (not plants; AC+ individuals, *n* = 13) were compared to C– individuals that had either not licked C+ individuals (C+C– or C–C+ treatment, *n* = 3) or had licked a C– individual (C–C– treatment, *n* = 7). To extract clerodanoids from these samples, individual adults and leaf material of *A. reptans* were frozen, lyophilised and homogenised. Samples were extracted in 130 μL ethyl acetate (LC–MS grade, VWR) for 10 min, centrifuged, and 120 μL of supernatant dried under reduced pressure. Dried extracts were resuspended in 90% methanol (LC–MS grade, VWR), filtered, and analysed using an ultra‐high performance liquid chromatograph (Dionex UltiMate 3000, Thermo Fisher Scientific, San José, CA, USA) coupled to a quadrupole time of flight mass spectrometer (compact, Bruker Daltonics, Bremen, Germany) in negative electrospray ionisation mode (see Supporting Information S1 for detailed settings). Candidate metabolites which may have been taken up (and metabolised) by the insects from the plants were identified by comparing the chromatograms of C+ and C– individuals, and by comparing extracts of leaves and all adults. The peak areas of two putative clerodanoids were manually integrated from the extracted ion chromatograms.

### 2.4 Analysis of Gene Expression

#### 2.4.1 RNAseq: Sample Collection, Library Preparation, Sequencing, and Alignment

Adult males (4–10 days old, *n* = 6 per treatment level C–, C+, AC+, total *n* = 18) were taken for transcriptome sequencing to assess the effects of the two types of clerodanoid acquisition. RNA was extracted using an Invitrogen PureLink RNA Mini Kit (ThermoFisher Scientific, Germany) with a DNase step (innuPREP DNase I Kit, analyticJena, Jena, Germany) and diluted to 25 μL using RNAse free water to a concentration of ~30–100 ng/μL per sample. Library preparation (rRNA removal via Ribo‐Zero) and sequencing using a NovaSeq6000 and S4 Flowcells (Illumina, CA, United States) was performed by Novogene (Cambridge, UK). Sequence quality was assessed using FastQC software version 0.11.9 (Andrews 2010) pre‐ and post‐cleaning of raw reads. Illumina specific adapter sequences, sequences shorter than 75 nucleotides, and poor‐quality sequences (quality < 4 over a 25 bp sliding window) were removed using Trimmomatic (Bolger et al. 2014). Reads retained after cleaning were mapped to the annotated *A. rosae* genome, AROS v.2.0 (GCA_000344095.2). Gene and transcript level counts were obtained with RSEM v1.3.1 (Li and Dewey 2011), using the rsem‐calculate‐expression script; mapping within this was performed with STAR v2.7.1a (Dobin et al. 2013).

#### 2.4.2 Differential Expression and Gene Set Enrichment Analysis

Differential expression (DE) analysis and the visualisation of results were carried out in R 4.0.2 (2020‐06‐22) using DESeq2 version 1.28.1 (Love et al. 2014). Counts were imported from the RSEM output to DESeq2 using Tximport version 1.16.1 (Soneson et al. 2015) to account for read length and summarise transcript‐level mapping for analysis at the gene‐level. Prior to DE analysis, genes with zero counts in all samples and those with low counts (< 10) in less than a quarter of samples were excluded from the dataset. One sample (AC+) was excluded from the analysis based on its large overall expression differences relative to all other samples (Gierliński et al. 2015). The predictor variable was modelled as ‘treatment’ with three different levels (C–, C+, AC+). We performed all three of the individual pairwise comparisons within the model built from all samples using a Wald test and shrunken LFC values with apeglm version 1.10.1 (Zhu et al. 2019). *p*‐Values were adjusted for multiple testing via the Benjamini‐Hochberg (Benjamini and Hochberg 1995) using a false discovery rate (FDR) of 0.05, resulting in a final list of significantly differentially expressed (DE) genes (both relatively up‐ or down‐regulated). Among these significantly DE genes we searched for the terms linked to genes with known roles in sequestration and detoxification pathways, using the keywords: ‘Cytochrome P450’, ‘Glycosidase’, ‘Glutathione’, ‘GST’, ‘Glucuronosyltransferase’, ‘Glycosyl transferase’, ‘UDP’, ‘Carboxy’, ‘Cholinesterase’, ‘CCE’, ‘CE’, ‘ATP binding’, ‘ABC transporter’, ‘Heat shock’, and ‘HSP’. Significantly DE genes were compared among contrasts in R using a Venn diagram (Venn.Diagram, v 1.6.20), and heatmaps were created of significant genes using the normalised counts scaled per gene (scale = ‘row’) (pheatmap, v 1.0.12).

To characterise DE at the pathway level, KEGG (Kyoto Encyclopedia of Genes and Genomes) terms were allocated to the gene expression data using annotations from the KEGG Automatic Annotation Server (KAAS; Moriya et al. 2007). A bi‐directional best hit KAAS was run using the predicted genes from the *A. rosae* genome, with 40 different insect species selected for reference (Supporting Information S2). Sixty‐five percent of the annotated genes for which we had read counts were assigned at least one KEGG term. As multiple genes matched to the same KEGG term, the normalised reads were summed across all genes that matched to each term. Furthermore, in the few cases (< 30) in which multiple KEGG terms were assigned to the same gene, the read counts were duplicated for each individual KEGG term. A gene set enrichment analysis (GSEA) was then carried out on the normalised counts using GAGE (Luo et al. 2009) and pathview (Luo and Brouwer 2013) in R, using an FDR‐adjusted *p*‐value cut‐off of < 0.05 to identify DE pathways.

### 2.5 Statistical Analysis

All data were analysed using R version 3.6.3 (R Development Core Team 2020). The alpha level was set at 0.05 for all tests and model residuals were checked for normality and variance homogeneity. Stepwise backwards deletion using Chi‐squared ratio tests (package: MASS, version 7.3–51.6) was employed to reach the minimum adequate model (Crawley 2012). Posthoc analyses were carried out using the package ‘multcomp’ (version 1.4‐13, Hothorn et al. 2008).

Differences in the occurrence (number of events) and duration of successful licking were assessed at both the treatment or contest level (C+ vs. C+; C+ vs. C–; C– vs. C–) and the level of the individual (focal individual = C+/C–, non‐focal individual = C+/C–). Variation in the occurrence of successful licking between the different treatment levels was tested using a negative binomial hurdle model to account for zero inflation (dist = glm.nb, link = logit, package: pscl version 1.5.5, Jackman et al. 2015), where licking occurrence (count) was the response and treatment the predictor variable in both the count and zero inflation parts of the model. Model fit was assessed using DHARMa package (version 0.3.2.0, Hartig 2020). A linear model (package: MASS) was used to assess differences between treatment levels in log‐transformed licking duration(s) for those assays in which licking occurred. Variation in the occurrence of successful licking based on focal and opponent clerodanoid exposure (C+ or C–) was tested using a negative binomial model to account for overdispersion (glm.nb, package: MASS) with focal and opponent clerodanoid exposure (C+ or C–) and their interaction as predictor variables. A linear model was used to assess differences between treatments in log licking duration (s) where focal and opponent clerodanoid exposure (C+ or C–), and their interaction were the predictor variables.

Binomial models (package: MASS, link = ‘logit’) were used to establish whether (a) licking *A. reptans* leaves and (b) licking conspecifics resulted in the acquisition of clerodanoids by *A. rosae* . The presence/absence of either of the two putative clerodanoid peaks from the chemical analyses was set as the response variable and either (a) focal individual clerodanoid status (C– or C+) or (b) non‐focal individual clerodanoid status (C– or C+) and conspecific licking (Yes/No) as the predictor variables. The relationship between licking duration and the square root of peak areas of each of the two metabolites identified as putative clerodanoids was tested using a linear model.

## 3 Results

### 3.1 Contest Behaviour

**FIGURE 2:**
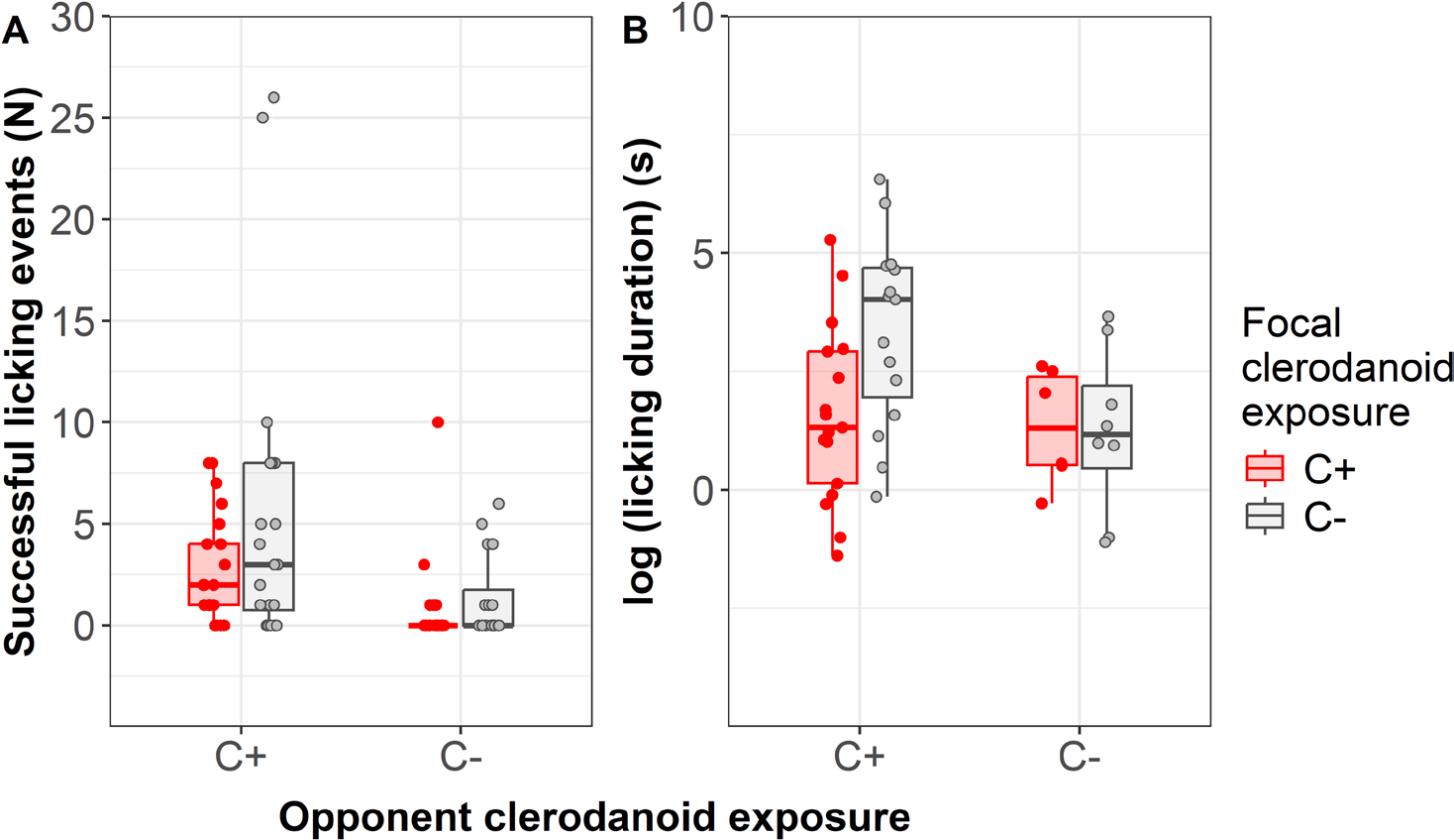
Effects of clerodanoid exposure of focal and opponent adult males of *Athalia rosae* (C+ = licked *Ajuga reptans* leaf, C– = not licked) on the licking behaviour of the focal individual on another male (20–23 replicates per treatment). (A) Number of successful licking events by the focal individual and (B) total duration of successful licking events. Boxes represent medians and interquartile ranges, whiskers extend to 1.5 times the interquartile ranges below the 25th percentiles or above the 75th percentiles, respectively.

The number of successful licking events was significantly higher in contest pairs where at least one individual was clerodanoid exposed (C+C+, C+C–) than in the control (C–C–) (*X* ^*2*^ = 18.951, *p* < 0.001; Table 1), with the clerodanoid status of both the focal (*X* ^*2*^_1,82_ = 4.058, *p* = 0.044) and opponent (non‐focal individual) (*X* ^*2*^_1,82_ = 16.154, *p* < 0.001) having a significant, but noninteractive effect (*X* ^*2*^_1,81_ = 0.006, *p* = 0.94) on licking occurrence. Focal individuals were more likely to lick the opponent when they were C– or the opponent was C+ (Figure 2A). The licking duration was also significantly affected by treatment (*F*_2,60_ = 5.801, *p* = 0.005), being longest in contest pairs in which one individual was clerodanoid‐exposed (‘C+C–’—‘C+C+’, *Z* = 2.658, *p* = 0.021; ‘C+C–’—‘C–C–’, *Z* = 2.800, *p* = 0.014), with the clerodanoid status of both the focal (*F*_1,43_ = 4.918, *p* = 0.032) and opponent individual (*F*_1,43_ = 4.406, *p* = 0.042) having a significant, but no interactive effect (*F*_1,42_ = 2.461, *p* = 0.124) on licking duration (Figure 2B).

**TABLE 1:** Results of zero‐inflated negative binomial regression (negative binomial hurdle model) testing the effect of treatment on the occurrence of successful licking behaviour in *Athalia rosae* contests.

Parameter	Estimate	pLL	pUL
Count (Intercept)	4.15	2.91	5.35
Count C+ vs. C−	2.06	1.42	3.11
Count C+ vs. C+	1.39	0.99	2.11
Zero (Intercept)	1.00	0.38	2.60
Zero C+ vs. C−	3.40	1.08	12.10
Zero C+ vs. C+	9.50	2.18	4.54E+05

*Note:* Estimate is the estimated incident risk ratio (IRR) for the negative binomial model (‘count’) and the odds ratio (OR) for the logistic (zero inflation) model (‘zero’). Bootstrapped percentile adjusted lower (pLL) and upper (pUL) confidence intervals are also given.

### 3.2 Pharmacophagy and Conspecific Transfer of Clerodanoids

Two putative clerodanoids were found in 75% of C+ individuals, with 529.23 and 527.21 *m*/*z*, representing the [M+HCOOH–H]^−^ adducts of the candidate features 482.22 *m*/*z* (C_24_H_34_O_10_) and 484.23 *m*/*z* (C_24_H_36_O_10_). The peaks of these features, hereafter referred to as compounds 482 and 484, appeared in high intensities only in individuals that had direct or indirect access to leaves, but not in the C– individuals or in the leaves.

**FIGURE 3:**
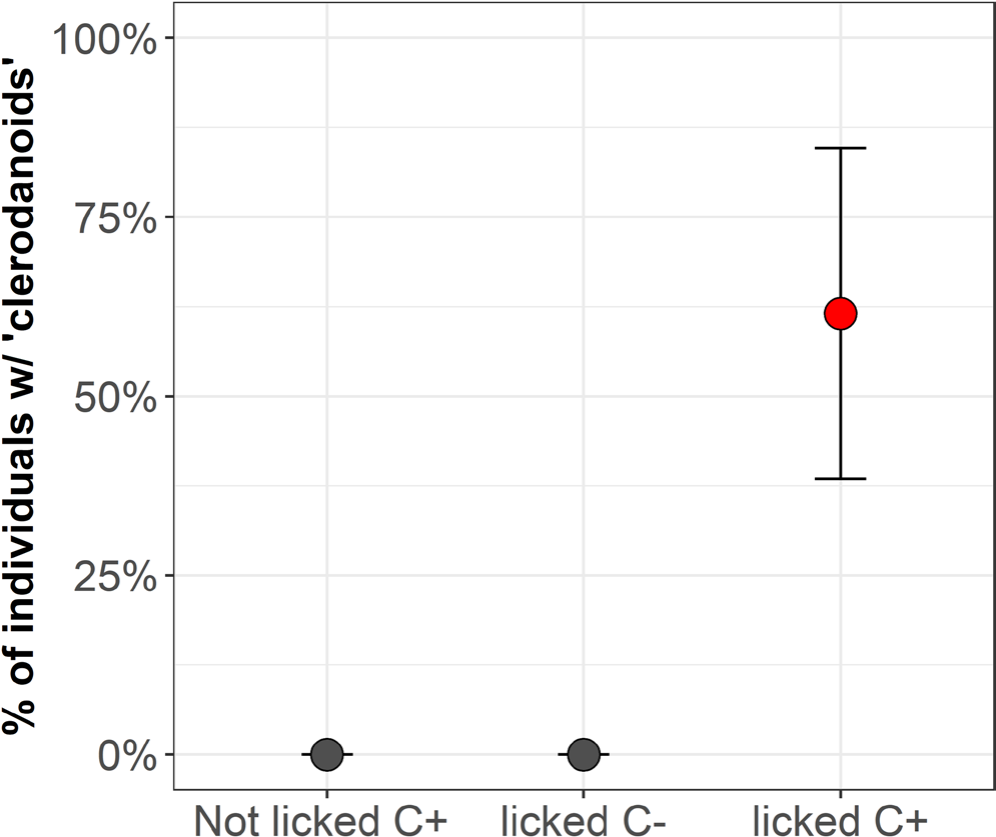
Percentage of C– males of *Athalia rosae*, in which clerodanoids (compounds 482 and 484) could be detected by chemical analysis after not licking a C+ individual (*n* = 3), licking a C– individual (*n* = 7), or licking a C+ individual (*n* = 13), turning them into AC+.

Licking leaves significantly influenced whether *A. rosae* individuals obtained the two clerodanoids (*X* ^*2*^_1,13_ = 11.193, *p* < 0.001). Licking by C– on C+ individuals significantly increased the likelihood of these individuals to obtain clerodanoids (AC+) compared to those that did not lick a C+ individual or that licked a C– individual (clerodanoid status of opponent: *X* ^*2*^_1,20_ = 9.597, *p* = 0.002; licking occurrence: *X* ^*2*^_1,20_ = 4.857, *p* = 0.027; Figure 3). However, there was no significant effect of licking duration by C– individuals on the amount of clerodanoids (= square‐root transformed peak area) that they acquired (compound 482: *F*_1,11_ = 1.745, *p* = 0.213; compound 484: *F*_1,11_ = 2.420, *p* = 0.148).

### 3.3 Comparison of Gene Expression in C–, C+ and AC+ Individuals

**FIGURE 4:**
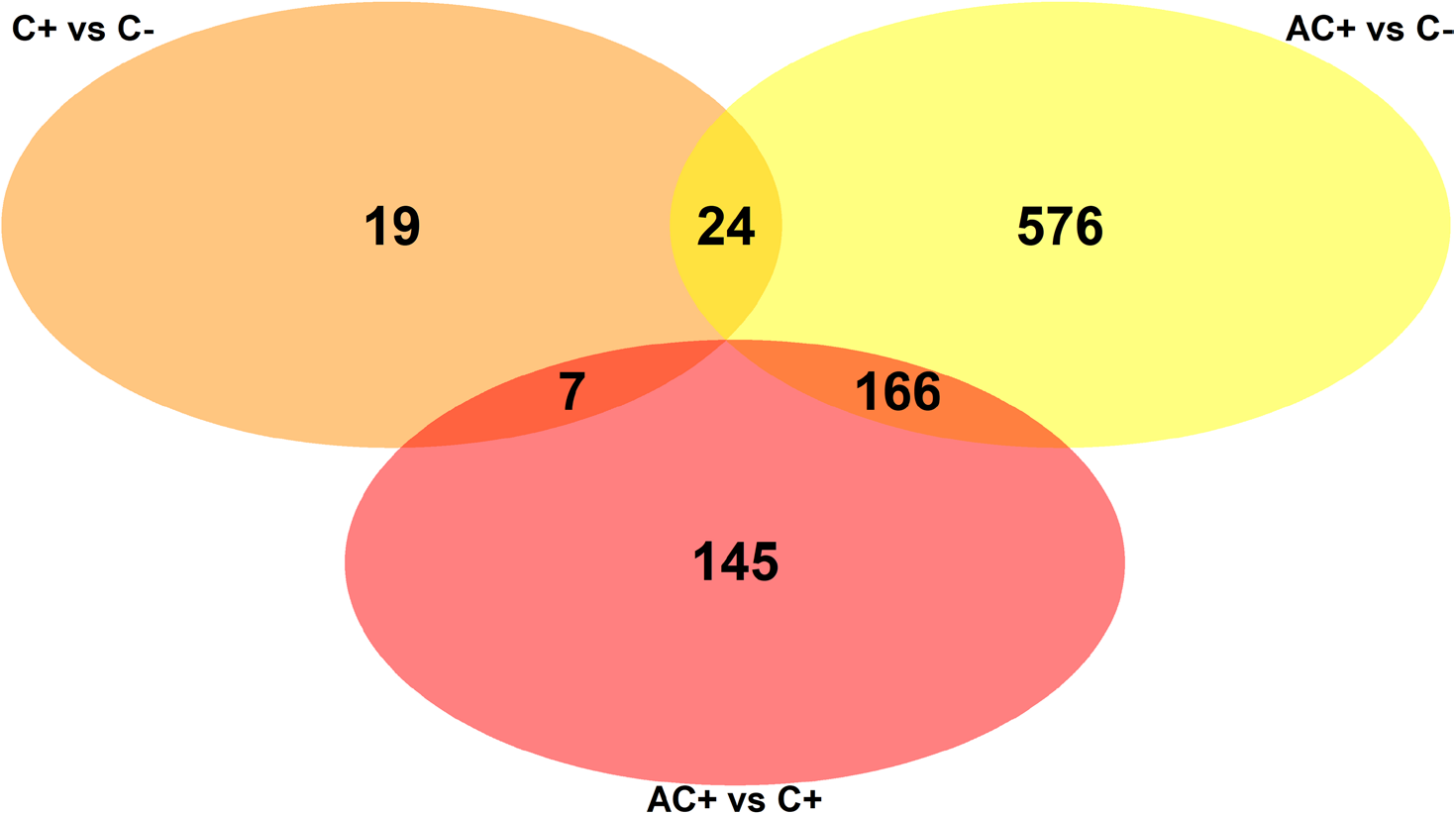
Venn diagram illustrating the number of unique and shared differentially expressed genes resulting from three pairwise comparisons (AC+ vs. C+, red; AC+ vs. C–, yellow; C+ vs. C–, orange), in which male *Athalia rosae* had access to clerodanoids via licking *Ajuga reptans* leaves (C+), via licking a conspecific C+ individual (AC+) or had no access to clerodanoids (C–).

Sequencing of all individuals yielded a total of 1.24 billion reads with an average of 68 million reads per sample (SEM: ±2 million reads) and an average GC content of 40%. More than 98% of reads were retained after cleaning. Prior to normalisation the average number of mapped reads per sample was 33,675,600; this represents on average 92% of the reads aligning to the reference genome (range 90%–93%). Access to clerodanoids via conspecific licking (AC+) had the most pronounced effect on gene expression (Figure 4, Supporting Information Figure S1). Between AC+ and C+ males, 318 genes and between AC+ and C– males, 766 genes were significantly DE (LFC > 0, adjusted *p* < 0.05). Considerably fewer (50) genes were DE between C+ and C– individuals (see Supporting Information S3 for full DE output). Across all tests, the maximum fold change ranged from 1.3 to 4.7×.

We identified DE genes that were shared between the AC+/C– and AC+/C+ contrasts, which may indicate genes linked to conspecific licking behaviour (166 DE genes, Figure 4, Supporting Information S4). The most pronouncedly upregulated gene in AC+ (fold change 4.5 in AC+/C– and 4.7× in AC+/C+) was the *takeout* gene (to/CG11853), linked to feeding and courtship behaviour in *Drosophila melanogaster* (Dauwalder et al. 2002; Sarov‐Blat et al. 2000). Also upregulated in AC+ was the *flightin* gene (fln/CG7445, fold change 1.4 in AC+/C– and 1.7× in AC+/C+), likely involved in the regulation of flight muscle contraction (Henkin et al. 2004). One of the genes shared by AC+ vs. C– and C+ vs. C– differential expression (24 DE genes, Figure 4, Supporting Information S4), which may indicate genes linked to pharmacophagy, was *tdc*, which encodes L‐tyrosine decarboxylase and is linked to stress responses, acid resistance and drug metabolism (Perez et al. 2015; van Kessel et al. 2019).

Among the DE genes, we identified putative members of previously identified canonical detoxification pathways, namely P450s, GSTs, UDPs, CE/CCEs, ABC transporters, and HSPs (Table 2). In the AC+/C– contrast, 11 of these genes were present (8 were higher in AC+). In the C+/C– contrast, there were 13 genes, all of which were more highly expressed in C+ individuals. These two contrasts shared 5 genes (2 HSP, 1 GST, 1UDP and 1 P450, Supporting Information S4), all of which were lower in C– individuals. With 38 putative detoxification genes, the AC+/C+ comparison had the highest number of putative detoxification genes, the most abundant of which were seven P450s and 14 UDPs (Supporting Information S4, Table 2). P450s were upregulated in AC+ relative to both C– (*n* = 2) and C+ (*n* = 7); different genes from this category were also upregulated in C+ relative to both AC+ (*n* = 2) and C– (*n* = 4). Of note, 14 UDPs were upregulated in AC+ relative to C+ (Table 2).

**TABLE 2:** Significantly DE genes belonging to canonical detoxification pathways, sampled in adult males of *A. rosae* from a given ‘treatment’ group.

	AC+ vs. C–		C+ vs. C–		AC+ vs. C+	
	Higher in AC+	Higher in C–	Higher in C+	Higher in C–	Higher in AC+	Higher in C+
Cytochrome P450	2	0	4	0	7	5
Glutathione S‐transferases (GSTs) and glycosidases	1	1	3	0	0	3
UDP‐glucuronosyltransferases and UDP‐glycosyltransferases (UDP)	2	1	3	0	14	2
Carboxy/cholinesterase (CEs and CCEs), including acetylcholinesterase	1	0	0	0	1	1
ABC transporters (ABCs)	0	0	0	0	1	1
Heat shock proteins (HSPs)	2	1	3	0	1	2

*Note:* Full details of these are listed in the far right column of Supporting Information S4 DE Full Results. AC+ = adult males of *A. rosae* that had access to clerodanoids via licking conspecifics, C+ = males that had access to clerodanoids via licking *A. reptans* leaves, and C– = those that had no access to clerodanoids.

GSEA using KEGG annotations revealed that AC+ individuals showed the highest number of differentially regulated pathways compared to individuals of the two other treatments and that many of these pathways were shared (Table 3; see Supporting Information S5 for full output). The pathways upregulated in AC+ fall roughly into three categories: energy and carbohydrate metabolism, endocrine systems, and circulatory systems. The cGMP‐PKG signalling pathway was also significantly upregulated in AC+ individuals compared to both C– and C+ individuals and plays a role in a number of processes including muscle contraction and memory formation in insects (Matsumoto et al. 2018). Furthermore, the focal adhesion pathway, the turnover of which increases with stress (Aedo et al. 2019), was significantly upregulated in AC+ compared to C– individuals. The ‘ribosome’ pathway was significantly downregulated in AC+ individuals in comparison to individuals of the two other treatments and also downregulated in C+ compared to C– adults.

**TABLE 3:** Significantly differentially expressed KEGG gene sets/pathways (p < 0.05) between adult males of *A. rosae* from a given ‘treatment’ group (left) vs. the ‘control’ (right).

Gene set/pathway	AC+ vs. C–	AC+ vs. C+	C+ vs. C–
Pentose phosphate pathway^a^	Up	—	—
Fructose and mannose metabolism^a^	Up	—	—
Oxidative phosphorylation^a^	Up	Up	—
Carbon metabolism^a^	Up	Up	—
Ribosome	Down	Down	Down
cGMP‐PKG signalling pathway	Up	Up	—
Cardiac muscle contraction^b^	Up	Up	—
Adrenergic signalling in cardiomyocytes^b^	Up	Up	—
Focal adhesion	Up	—	—
Thyroid hormone signalling pathway^c^	Up	Up	—
Glucagon signalling pathway^c^	Up	—	—

*Note:* AC+ = adult males of *A. rosae* that had access to clerodanoids via licking conspecifics, C+ = males that had access to clerodanoids via licking *A. reptans* leaves, and C– = those that had no access to clerodanoids. The pathways are grouped into three categories.

^a^ Energy and carbohydrate metabolism.

^b^ Endocrine systems.

^c^ Circulatory systems.

## 4 Discussion

Here we investigated a special form of pharmacophagy in adults of the sawfly *A. rosae* and demonstrated that individuals can acquire metabolites known to influence defence and mating behaviour (Amano et al. 1999; Paul and Müller 2022; Singh et al. 2022) not only from plants but also exclusively from the bodies of conspecifics that had previously fed on those plants. In contrast to plant‐derived nuptial gifts, which are usually transferred from males to females in some insect species (Wink 2019; Zagrobelny et al. 2007), plant‐derived chemicals are transferred within and between both sexes in this sawfly species predominantly in the context of dyadic contest behaviour (this study and Paul and Müller 2022). This behaviour may become particularly important if direct access to clerodanoid‐containing plants is temporally or spatially limited, for example, due to patchiness or seasonality. Indeed, consistent with this, wild‐caught *A. rosae* adults show substantial intraspecific variation in clerodanoids, including individuals with no detectable clerodanoids (Singh et al. 2024).

The agonistic licking interactions recorded here between *A. rosae* individuals likely entail costs generally associated with fighting, such as increased energy expenditure (Hack 1997), risk of injury (Lane and Briffa 2017), and predation (Baker et al. 1999). We therefore predicted that, as individuals were size matched (proxy for RHP), the value of the resource (RV; Arnott and Elwood 2009; Stockermans and Hardy 2013), that is, having clerodanoids, determines the likelihood of agonistic licking an opponent. Indeed, C– individuals were more likely to successfully lick their opponents. However, contrary to our predictions, C+ individuals continued to lick opponents, even when those opponents were also C+. Furthermore, comparison of gene expression data revealed an upregulation of pathways linked to increased energetic expenditure and metabolic stress in AC+ individuals; this supports the hypothesis that agonistic licking behaviour is energetically costly. These behavioural results indicate the high value of accruing clerodanoids even in the face of significant fighting costs. Acquiring clerodanoids from conspecifics as opposed to directly from plants may carry the additional benefit that these clerodanoids are already metabolised compared to the compounds taken up from the plants. They also suggest that the beneficial effects of clerodanoids might be concentration‐dependent, a common attribute of specialised metabolites in insect defence (Burdfield‐Steel et al. 2019; Ode and Ghosh 2025). A similar phenomenon of kleptopharmacophagy has been observed in milkweed butterflies, where adult butterflies scratch living caterpillars and potentially take up alkaloid‐containing fluid from them (Tea et al. 2021).

The putative clerodanoids found in C+ individuals were not present in *A. reptans* leaves, indicating that the plant clerodanoids are further metabolised by the sawflies, similar to findings of previous work on *A. rosae ruficornis* (Nishida et al. 2004) and as already reported for *A. rosae* (Brueggemann et al. 2023). Many phytochemicals are metabolised by insects after uptake to reduce their toxicity and increase their solubility (Erb and Robert 2016). Only a small number of canonical detoxification genes were found upregulated in C+ individuals of *A. rosae* , but those that were present were associated with metabolism of toxins. Upregulation of such genes may be rapidly induced when the uptake of specialised metabolites is initiated but may also level down rapidly, leading to the observed small response. Alternatively, although shown to be distasteful (Nishida and Fukami 1990), the modified clerodanoids might not actually be toxic or may simply require other pathways and genes for their processing. It is still unclear to what extent clerodanoids are ingested by *A. rosae*. Plant clerodanoids may instead be collected using the mouthparts, metabolised by salivary glands (as seen in species of Hemiptera, e.g., Zhu et al. 2016), or broken down nonenzymatically and then be redistributed over the body via grooming. The presence of clerodanoid compounds in surface extracts of *A. rosae* adults after licking *A. reptans* leaves (Brueggemann et al. 2023) supports this possibility.

Our findings suggest that the metabolic costs of clerodanoid pharmacophagy from plants in *A. rosae* are low, at least at the time point when samples were taken. This is consistent with the broader body of research on sequestration costs, which are generally negligible (Zvereva and Kozlov 2016). In contrast, the metabolic costs of acquiring clerodanoids from conspecifics rather than plants appear to be higher, as illustrated by the upregulation of gene pathways related to carbon metabolism and ATP production in AC+ individuals, and not in the C+ individuals. These costs could also be related to increased activity associated with acquiring clerodanoids, but this seems unlikely because it also takes activity to acquire them directly from the plant. Although we have not directly linked these metabolic costs to fitness consequences, the fact that individuals are willing to bear the potential costs of fighting for clerodanoids, even when they already possess some, indicates that the fitness benefits of clerodanoid acquisition outweigh these costs. Moreover, reduced survival was observed when individuals were ‘licked’, suggesting that the agonistic interactions may indeed have a fitness cost (Singh et al. 2024).

In AC+ individuals we also observed upregulation of more putative detoxification genes than in the other treatments, with the most numerous being cytochrome P450s and UDPs. Cytochrome P450 genes were upregulated compared to both C– and C+ individuals. P450s often are involved in phase 1 metabolism of plant specialised metabolites through oxidation, reduction, or hydrolysis of metabolites (Birnbaum and Abbot 2020). It is noteworthy that different P450 genes were also upregulated in C+ relative to both AC+ and C–, suggesting that metabolism‐related detoxification generally occurs in response to clerodanoids. UDPs were also upregulated in AC+ relative to both C– and C+ individuals and were the most abundant putative detoxification gene family that we identified. These genes act by adding sugar groups to water insoluble molecules, making them more hydrophilic and thus more easily excreted (Ahn et al. 2012). Together, the upregulation of these genes suggests that upregulation of metabolic pathways may be primarily associated with detoxification activities. The specific functions of these genes in *A. rosae* require further investigation.

In addition to the upregulation of gene pathways related to the increased metabolic demands caused by fighting in AC+ individuals, the expression of ribosomal proteins (RPs) was downregulated in AC+ males compared to both C+ and C– individuals, following the pattern AC+ < C+ < C–. The expression of genes and gene pathways responsible for RPs is likely not as stable as previously assumed, with their differential expression being recorded in response to a number of factors such as heat stress (Hales et al. 2017; Paraskevopoulou et al. 2020) or the ingestion of ribosomal inactivating proteins (Celorio‐Mancera et al. 2013; Stirpe 2004). RPs play numerous roles outside of ribosomal biogenesis (Zhou et al. 2015); for example, RP expression might be lower in AC+ and C+ individuals because of their role in innate immune response (Mukhopadhyay et al. 2008, 2009). In monarch butterflies ( *Danaus plexippus* ), the acquisition of plant metabolites may help defend against parasites whilst resulting in the downregulation of several genes that play a role in immunity (Tan et al. 2019). The acquisition of clerodanoids may likewise increase general immunity due to potential antimicrobial effects (Bozov et al. 2015; Zanchi et al. 2021), leading to the downregulation of genes involved in innate immunity including ribosomal proteins. Additionally, the more pronounced downregulation of RPs in AC+ individuals (when compared to both C+ and C–) may reflect the sensitivity of ribosome biogenesis to the energy status of the cell (Zhou et al. 2015), thus conserving ATP.

In conclusion, we show that pharmacophagy involves not only the uptake of plant compounds but also their subsequent transfer among conspecifics. The acquisition of specialised plant metabolites alters an individual's metabotype, influencing its social interactions. Gene expression data indicates that clerodanoid sequestration from plants incurs low metabolic costs, whereas acquiring clerodanoids exclusively from conspecifics is metabolically costly and elicits a higher detoxification response. Nevertheless, conspecifics are willing to bear these costs, even if they have already acquired clerodanoids from plants.

## Author Contributions

Conceptualisation: Sarah C. Paul and Caroline Müller. Methodology: Sarah C. Paul, Alice B. Dennis and Lisa Johanna Tewes. Investigation: Sarah C. Paul, Lisa Johanna Tewes and Jeanne Friedrichs. Resources: Caroline Müller, Alice B. Dennis. Formal analysis and validation: Sarah C. Paul, Alice B. Dennis, Lisa Johanna Tewes and Pragya Singh. Data curation management: Sarah C. Paul, Lisa Johanna Tewes and Pragya Singh. Writing: Sarah C. Paul and Caroline Müller. Alice B. Dennis, Lisa Johanna Tewes and Pragya Singh contributed to the final version.

## Funding

The project was funded by the German Research Foundation (DFG: https://www.dfg.de/) as part of the SFB TRR 212 (NC^3^). This work was supported by Deutsche Forschungsgemeinschaft, Project 396777467 (granted to CM).

## Ethics Statement

Research was carried out following the ASAB/ABS Guidelines for the Use of Animals in Research (Animal Behaviour, 2020, 159, I–XI). Field collections were carried out using a sweep net to catch whilst feeding on flowers or whilst in flight, eliminating excess bycatch (approx < 0.1% of catch). After the behavioural assays those individuals not frozen for use in chemical or gene expression analyses were returned to the *A. rosae* stock culture. All the research complies with applicable laws on sampling from natural populations and animal experimentation (including the ARRIVE guidelines).

## Conflicts of Interest

The authors declare no conflicts of interest.

## Supporting information


**Figure S1:** Heatmap showing the expression of genes (scaled normalised counts) across all samples for genes that were significantly (sig) differentially expressed (DE) between (A) AC+ and C– individuals (766 genes), (B) AC+ and C+ (318 genes), and (C) C+ and C– (50 genes). AC+ = adult males of *Athalia rosae* that had access to clerodanoids via nibbling on conspecifics, C+ = males that had access to clerodanoids via nibbling on *Ajuga reptans* leaves, and C– = those that had no access to clerodanoids.

**Data S1:** UHPLC‐QTOF‐MS/MS Methods Settings.

**Data S2:** Species chosen for KAAS BLAST.

**Data S3:** DE Full results.

**Data S4:** DE genes overlap Tables.

**Data S5:** KEGG results.

## Data Availability

Data and code for behavioural analysis and for gene expression analysis are accessible via ZENODO at https://doi.org/10.5281/zenodo.17086244. Raw Reads: BioProject ID = PRJNA700827. BioSample accession numbers = SAMN17839831‐48 (http://www.ncbi.nlm.nih.gov/bioproject/700827).
