# Supplementary material for: Fighting for Chemicals: Pharmacophagy‐Induced Contest Behaviour and Its Molecular Signatures in a Sawfly
Source: Mol Ecol. 2026 Apr 15;35(8):e70345. doi: 10.1111/mec.70345 (PMC13080588; doi:10.1111/mec.70345)
Supplement: Supplementary file 1 — Figure S1: Heatmap showing the expression of genes (scaled normalised counts) across all samples for genes that were significantly (sig) differentially expressed (DE) between (A) AC+ and C– individuals (766 genes), (B) AC+ and C+ (318 genes), and (C) C+ and C– (50 genes). AC+ = adult males of Athalia rosae that had access to clerodanoids via nibbling on conspecifics, C+ = males that had access to clerodanoids via nibbling on Ajuga reptans leaves, and C– = those that had no access to clerodanoids. [file MEC-35-e70345-s004.docx]

**Supplementary material**

**S1: UHPLC-QTOF-MS/MS settings**

Extracts were separated on a Kinetex XB-C18 column (1.7 µm, 150 × 2.1 mm, with guard column, Phenomenex) at 45 °C on a gradient from 0.1% formic acid (p.a., eluent additive for LC-MS, ~98 %, Sigma-Aldrich; in millipore water) to 0.1 % formic acid in acetonitrile (LC-MS grade, Fisher Scientific, Loughborough, UK; eluent B) at a flow rate of 0.5 ml min^-1^. The proportion of eluent B started at 2%, increased to 30% B within 20 min, and further to 75% B within 9 min. Electrospray ionisation was done with the source settings: end plate offset: 500 V, capillary voltage: 3000 V, nebuliser (N_2_) pressure: 3 bar, dry gas (N_2_) flow and temperature: 12 l min^-1^ at 275 °C. Line spectra (50-1300 *m*/*z*) were acquired at a spectra rate of 1 Hz. A Na(HCOO)-based calibration solution was introduced to the source at the end of each sample for mass axis recalibration. The quadrupole settings were: ion energy: 4 eV, low mass: 90 *m*/*z*, and the collision cell settings were: collision energy: 7 eV, transfer time: 100 µs, pre-pulse storage: 5 µs. Chromatograms were processed using the software Compass Data Analysis 4.4 (Bruker Daltonics, Bremen, Germany).

**
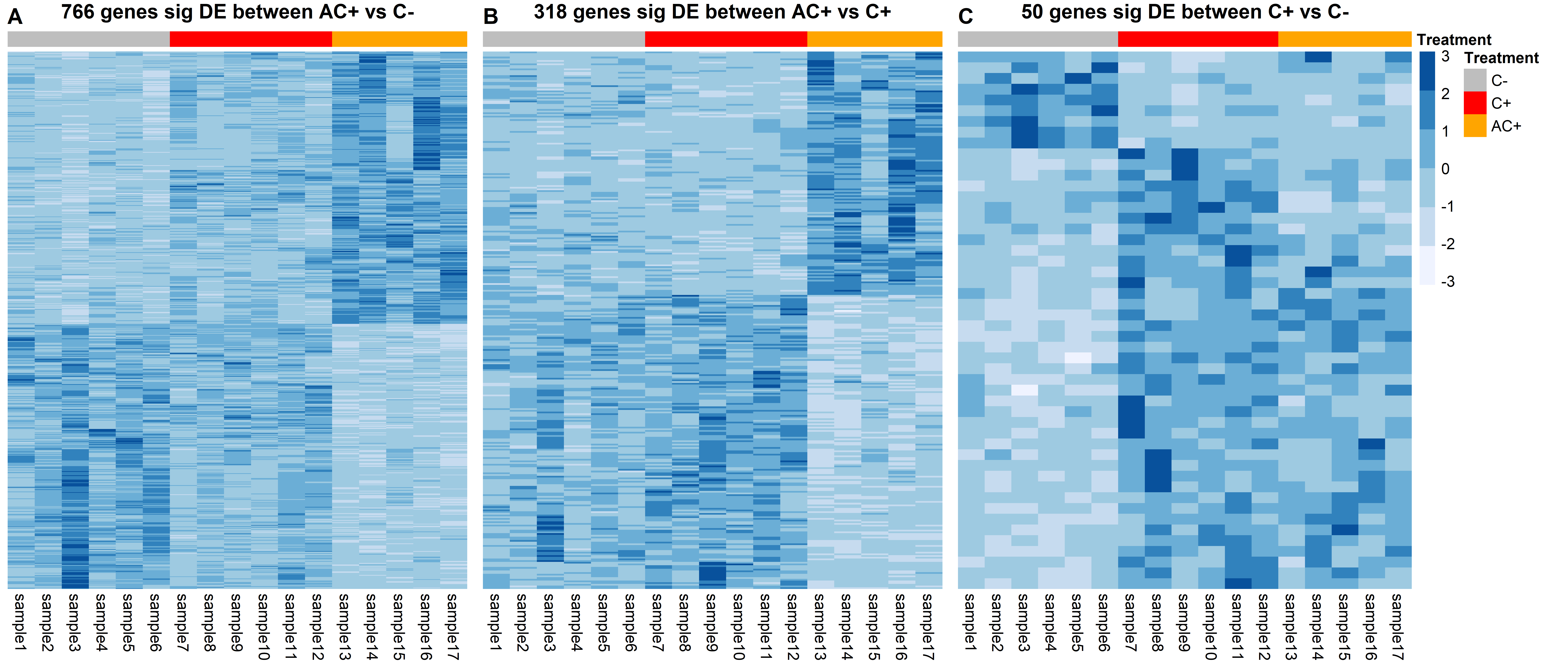
**

**Fig. S1:** Heatmap showing the expression of genes (scaled normalised counts) across all samples for genes that were significantly (sig) differentially expressed (DE) between A) AC+ and C- individuals (766 genes), B) AC+ and C+ (318 genes), and C) C+ and C- (50 genes). AC+ = adult males of *Athalia rosae* that had access to clerodanoids via nibbling on conspecifics, C+ = males that had access to clerodanoids via nibbling on *A. reptans* leaves*,* and C- = those that had no access to clerodanoids.
